# Alternation between different types of evidence attenuates judgments of severity

**DOI:** 10.1371/journal.pone.0180585

**Published:** 2017-07-06

**Authors:** Jennifer C. Whitman, Jiaying Zhao, Rebecca M. Todd

**Affiliations:** 1 Department of Psychology, Northwestern University, Evanston, IL, United States of America; 2 Department of Psychology, University of British Columbia, Vancouver, BC, Canada; 3 Institute for Resources, Environment and Sustainability, University of British Columbia, Vancouver, BC, Canada; University of North Carolina at Chapel Hill, UNITED STATES

## Abstract

Most real-world judgments and decisions require the consideration of multiple types of evidence. For example, judging the severity of environmental damage, medical illness, or negative economic trends often involves tracking and integrating evidence from multiple sources (i.e. different natural disasters, physical symptoms, or financial indicators). We hypothesized that the requirement to track and integrate across distinct types of evidence would affect severity judgments of multifaceted problems, compared to simpler problems. To test this, we used scenarios depicting crop damage. Each scenario involved either two event types (i.e. mold damage and insect damage), or one event type. Participants judged the quality of the crop following each scenario. In Experiments 1 and 2, subjective judgments were attenuated if the scenario depicted multiple event types, relative to scenarios depicting single event types. This was evident as a shallower slope of subjective severity ratings, as a function of objectively quantifiable severity, for scenarios with multiple event types. In Experiment 3, we asked whether alternation between event types might contribute to this attenuation. Each scenario contained two event types, and the sequence of events either alternated frequently between types or was organized into two sequential groups. Subjective judgments were attenuated for scenarios with frequently alternating sequences. The results demonstrate that alternation between distinct event types attenuates subjective judgments of severity. This suggests that a requirement to integrate evidence across multiple sources places extra demands on the cognitive system, which reduces the perceived evidence strength.

## Introduction

When individuals judge the severity of a problem or the effectiveness of a solution, they often must integrate evidence over time. An extensive body of work on probabilistic reasoning has examined how people form and update beliefs while accumulating new evidence [1–3]. This work has often focused on how people accumulate a single type of evidence, such as the color of the beads drawn from a jar, the color of fish drawn from a lake, or monetary gains vs. losses [4–7]. Such paradigms allow the researcher to precisely match conditions for objectively quantifiable evidence strength while studying how evidence is integrated across time [8]. Here, we ask how judgments are affected by a need to track and integrate across multiple types of evidence while controlling for objective evidence strength.

Integrating across evidence types is essential for accurately judging the severities of many real-world problems. For example, in judging the severity of an illness, people need to consider the frequencies of several distinct symptoms. Another example is recognition of climate change impacts, which requires combining observations of droughts, floods, forest fires, and storms. Assessing many other environmental, economic, political, and social issues similarly requires integration across multiple distinct indicators. In each of these cases, judging a multifaceted problem requires tracking several distinct types of evidence and integrating them appropriately. These added requirements could increase the difficulty of accurately judging problem severity (relative to problems characterized by only one type of evidence). Here, we asked whether judgments of problem severity were attenuated by the involvement of more than one type of evidence.

In the current series of experiments, we manipulated the number of evidence types while controlling for evidence strength by presenting scenarios that each depicted twelve years of orchard crops. Each year depicted damage by either insects or mold. Each scenario depicted a different farmer trying a new breed of fruit in order to minimize damage. At the end of each scenario, participants judged how ‘good’ or ‘bad’ the crops had been on a Likert scale. Scenarios with two event types included six years with insect problems and six years with mold problems. Scenarios with one event type included either twelve years with insect problems or twelve years with mold problems. We hypothesized that integration across distinct types of events would affect severity judgment, by either attenuating or enhancing perceived evidence strength.

## Experiment 1

The goal of Experiment 1 was to test whether the judged severity of a problem would differ as a function of whether it involved two types of negative event or only one. We chose farming scenarios for this study (rather than scenarios depicting controversial or polarizing problems involving economics, politics or the environment). In addition to being optimal for within-subjects comparisons, the farming scenarios allowed us to match scenarios with one event type to those with two event types in terms of objective evidence strength. Evidence strength was operationalized as the percentage of damaged fruit, averaged across years, in each series of orchard crops.

### Materials and methods

#### Participants

Thirty-one undergraduate university students (*N* = 22 females, mean age of 20.4, *SD* = 4.4) at the University of British Columbia participated in the Experiment in exchange for psychology course credit. Ethics approval was obtained from the University of British Columbia Behavioural Research Ethics Board. All participants provided written informed consent.

#### Stimuli

Stimuli consisted of individual tree images, each of which served as an icon representing the crops of an entire orchard for one year. Each tree had twelve fruit, and each depicted damage by either insects or mold–never a mixture of both at once. From here on, we will refer to the years depicted by a tree with three damaged fruit as involving mild damage. A tree with six damaged fruit will correspond to moderate damage, nine damaged fruit will correspond to severe damage, and twelve damaged fruit to very severe damage. We also varied the locations of the damaged fruit by producing six possible versions of each tree image depicting damage to a given percentage of the fruit.

#### Procedure

**Fig 1:**
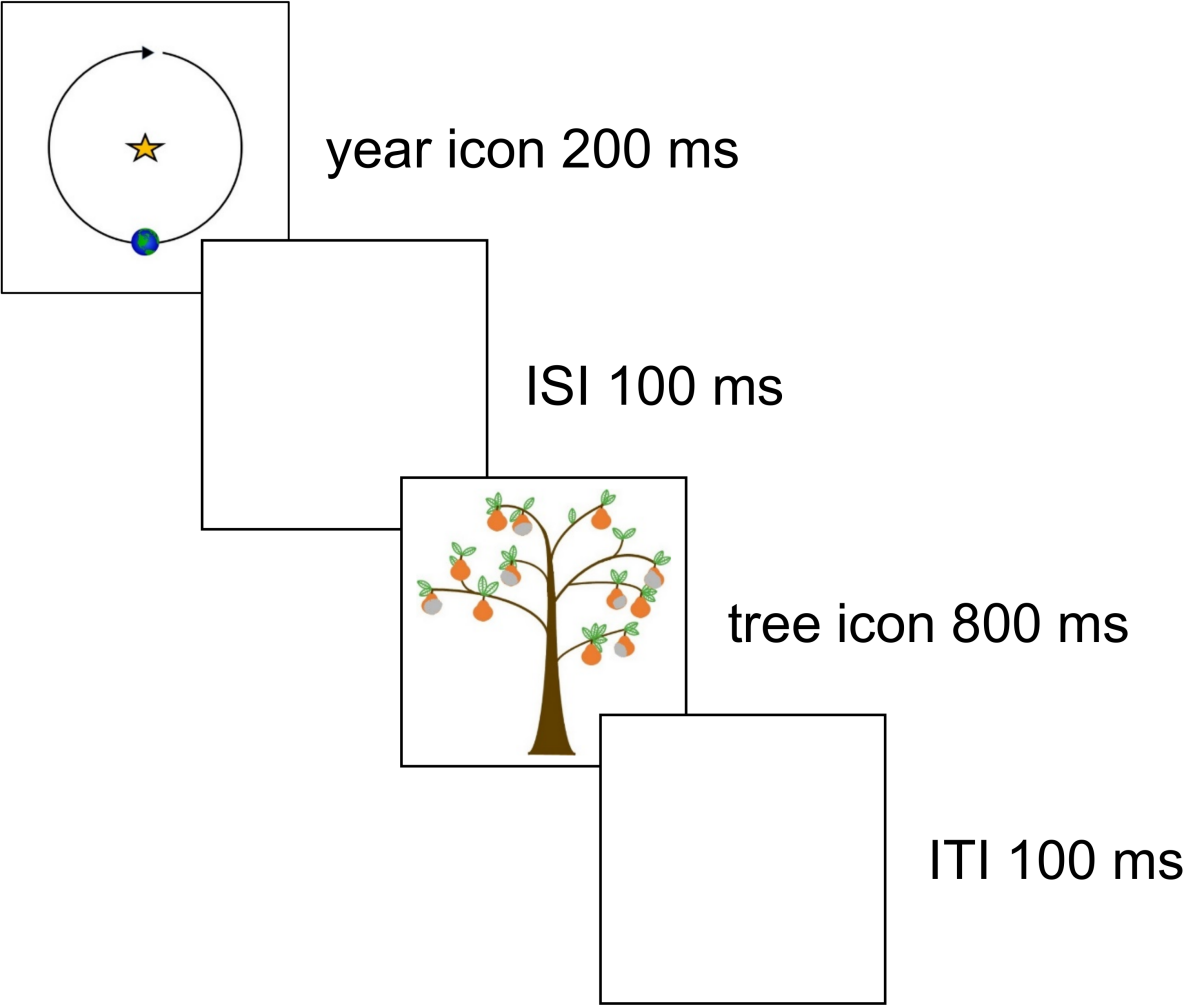
Sequence of events representing a single year within a twelve-year scenario.

Each participant judged how good vs. bad each series of orchard crops was in a total of forty scenarios. Each scenario involved a new farmer specified by a unique name. The farmer was said to be trying a new breed of pear in the hopes of it being more resistant to insects and mold. The scenario depicted twelve years of crops–thus, the participant would be judging the overall effectiveness of growing that new breed of pear across the twelve years. Each individual year was represented by the following sequence of events, also depicted in Fig 1. An image of a planet circling a star was presented for 200 ms (representing a new year), a blank inter-stimulus interval (ISI) lasting 100 ms, an image of a single pear tree was presented for 800 ms, then a blank inter-trial interval (ITI) lasted 800 ms. Following each tree image, the participant pressed the left mouse button if there had been insects on some of the fruit, or the right mouse button if there had been mold, as quickly and accurately as possible. After twelve years had been depicted, the participant rated how good or bad the crops had been by moving a cursor on a Likert scale. This was vertical and 320 pixels long, with the label ‘very good’ at the top and ‘very bad’ at the bottom.

Each twelve-year period involved either a relatively good series of crops or a relatively bad series. In a good series of crops, the twelve years were equally split between years with mild, moderate, and severe damage. This corresponded to 50% of fruit being damaged, averaged across the twelve years in a scenario. In a bad series of crops, the twelve years were equally split between years with moderate, severe, and very severe damage. This corresponded to 75% of fruit being damaged, on average. These levels of average severity formed one factor in our experimental design.

The other factor in our design was whether the scenario involved one or two event types. If there was one event type, then either all twelve years involved insect damage or all twelve years involved mold damage. If there were two event types, then the scenario involved six years with insect damage and six years with mold damage. In this case, the order of events was pseudo-randomized so that there were never more than two years in a row with the same event type. In other words, there were never three years in a row with mold problems or three years in a row with insect problems (in the condition with two event types). We took this approach of tightly controlling the rate of alternation, rather than using completely random sequences which would include both short and long streaks, because we expected alternation between event types to play a key role in integrating evidence for multifaceted problems. In Experiment 3, we explicitly manipulate alternation rate. In Experiments 1 and 2, we manipulate number of event types. The ordering of event severities was controlled by yoking scenarios between conditions. Specifically, for each scenario involving one event type and a relatively good series of crops, there was a matched scenario involving two event types and an equally good series of crops. Analogous yoked pairs were created for each relatively bad series of crops. These pairs were matched in terms of the ordering of the years with mild, moderate, severe, and very severe crop damage. Note that the two types of scenarios were equivalent in terms of the amount of information presented. A given scenario in the condition with two event types consisted of the presentation of 12 fruit trees, each of which contained 12 fruit, for a total of 144 fruit in total. A given scenario in the condition with one event type also involved the presentation of 144 fruit in total, also across 12 successively presented fruit trees. In sum, we matched the two-event-types condition with the single-event-type condition in terms of the objective frequencies of events.

Participants also completed four practice scenarios prior to the beginning of the main experiment. Each involved three years of each type (mild, moderate, severe, or very severe damage). One practice scenario involved twelve years of mold damage, one involved twelve years of insect damage, and the other two each combined six years of mold damage with six of insect damage.

#### Data analysis

In the Analyses of Variance reported in the current experiment and in all experiments below, we made Greenhouse-Geisser corrections for violation of the sphericity assumption, as implemented in SPSS software, when appropriate. These corrections ensure that the threshold for statistical significance is appropriate when variance is not uniform across pairs of conditions.

### Results and discussion

Data from the current experiment and all subsequent experiments in this paper will be made available on figshare.com (https://figshare.com/s/c18cd0ea9737508149c9). Four of the participants were excluded from the analysis because they failed to rate the scenarios with the least crop damage (‘good’ series) as better than the scenarios with the most crop damage (‘bad’ series). The remaining twenty-seven participants (*N* = 20 females) had a mean age of 19.7 years (*SD* = 1.5).

**Fig 2:**
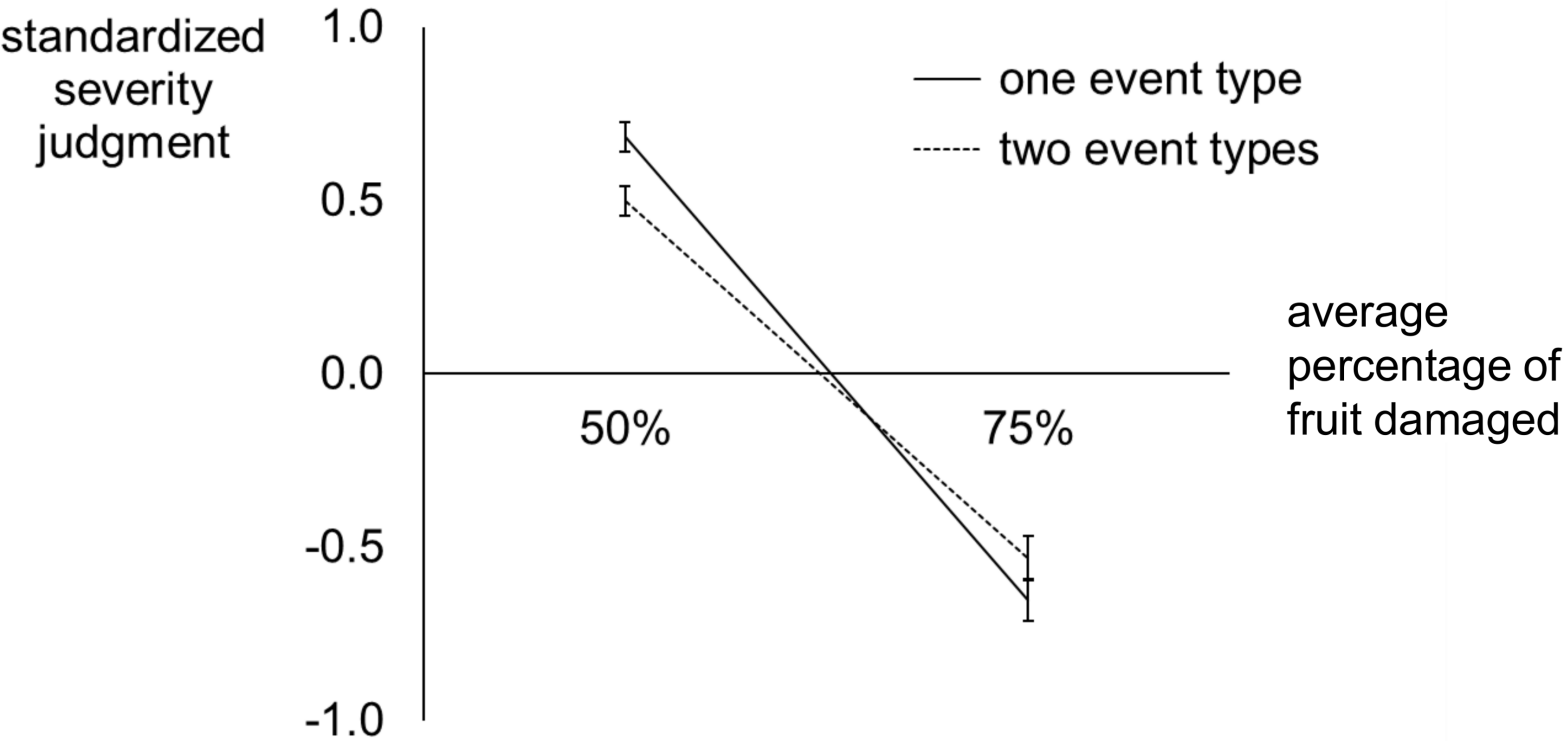
Experiment 1: Standardized subjective ratings of crop damage severity plotted as a function of objective crop damage severity (percentage of fruit damaged), plotted separately for scenarios involving only one event type across all twelve years and scenarios depicting two event types (six years with mold damage, six years with insect damage, order pseudo-randomized).

The main dependent variable in our analyses was the subjective rating, made at the end of each scenario, of how good or bad the series of crops was. Within each participant’s data, we transformed each individual rating to a standardized score by subtracting the mean of all ratings made (regardless of condition) and dividing by their standard deviation. These standardized subjective ratings of damage severity are plotted as a function of objective damage severity in Fig 2, separately for the scenarios with one and two event types.

We submitted the standardized ratings to a 2 × 2 repeated measures ANOVA with factors of Number of Event Types (one vs. two) and Damage Severity (the percentage of fruit damaged, by either mold or insects, averaged across all twelve years in a scenario). We found no main effect of Number of Event Types, *F*(1,26) = 0.25, *p* = .62, *η*^*2*^ = .01. There was a main effect of Damage Severity, *F*(1,26) = 295.67, *p* < .001, *η*^*2*^ = .92, and an interaction of Damage Severity with Number of Event Types, *F*(1,26) = 9.08, *p* = .006, *η*^*2*^ = .26. Planned contrasts (Bonferroni corrected) showed that judgments differed significantly as a function of number of events for the relatively good scenarios, *p* = .001, but not the relatively bad scenarios, *p* = .23. The simple main effect of Number of Event Types was stronger for the relatively good scenarios than for the relatively bad scenarios, *t*(26) = 3.01, *p* = .01.

Our finding of a significant interaction of Number of Event Types with Damage Severity is consistent with our hypothesis that the effect of evidence strength on severity judgments is influenced by the number of event types that must be considered. Here our findings indicate that severity judgments are attenuated when integration across multiple types of evidence is required. That pattern is illustrated by the shallower slope, visible in Fig 2, for subjective severity ratings plotted as a function of objective severity, when scenarios involved two event types. The fact that these conditions differed when 50% of fruit were damaged but not when 75% of fruit were damaged leads us to ask whether we could describe this interaction more clearly if there were more levels of objective severity. We tested this in Experiment 2.

## Experiment 2

The goal of Experiment 2 was to replicate the interaction found in Experiment 1 using a wider range of objective problem severities. This allowed us to further test our hypothesis that integration of distinct event types affects severity judgments. We expected the results of Experiment 1 to replicate the shallower slope for subjective severity, plotted as a function of objective severity, visible in the results of Experiment 1.

### Materials and methods

The methods of Experiment 2 were identical to those of Experiment 1 with the following exceptions.

#### Participants

Thirty-seven undergraduate university students participated in the experiment. Three participants were excluded because their subjective ratings of damage severity demonstrated a failure to distinguish between objective differences. More specifically, they were rejected if, in either the condition with one event type or the condition with two event types, the slope of their ratings of subjective severity as a function of objective severity was less than or equal to zero. The remaining thirty-four participants (*N* = 29 females) had a mean age of 19.8 years (*SD* = 2.9).

#### Procedure

The procedure for Experiment 2 was identical to that of Experiment 1 with the following exceptions. There were no years with very severe damage (corresponding to icons with twelve damaged fruit). This was an integral step in designing a study with four rather than two levels of severity. The four scenarios in the practice session each consisted of four years with mild damage, four with moderate damage, and four with severe damage. The main experiment involved eighty scenarios. These were evenly split into four levels of average damage severity. The scenarios with the worst series of crops consisted of eight years with severe fruit damage, two years with moderate fruit damage, and two years with mild fruit damage. The scenarios with the second worst crops consisted of six years with severe damage, four with moderate damage, and two with mild damage. The scenarios with the third worst crops consisted of two years with severe damage, four with moderate damage, and six with mild damage. Finally, the scenarios with the best crops consisted of two years with severe damage, two with moderate damage, and eight with mild damage. In other words, there were four discrete levels of objective severity. Either 63%, 58%, 42%, or 38% of the fruit were damaged, averaged across the twelve years in a scenario.

## Results and discussion

**Fig 3:**
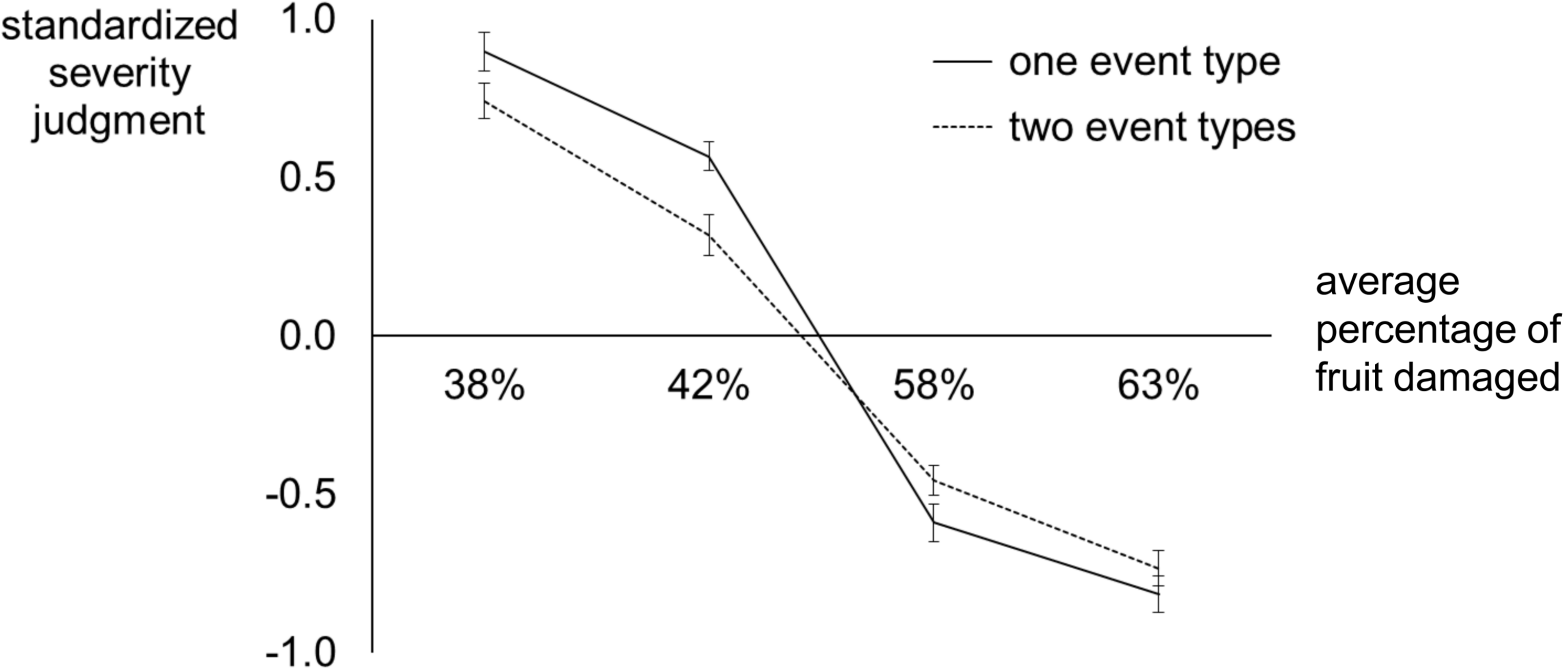
Experiment 2: Standardized subjective ratings of crop damage severity plotted as a function of objective crop damage severity (percentage of fruit damaged), plotted separately for scenarios involving only one event type across all twelve years and scenarios depicting two event types.

The standardized subjective ratings of damage severity are plotted as a function of objective damage severity in Fig 3. As in Experiment 1, we submitted these standardized ratings to a 2 × 2 repeated measures ANOVA with factors of Number of Event Types and Damage Severity. As in Experiment 1, we found no main effect of Number of Event Types, *F*(1,33) = 3.09, *p* = .09, *η*^*2*^ = .09. Again there was a main effect of Damage Severity, *F*(3,99) = 225.03, *p* < .001, *η*^*2*^ = .87, and an interaction of Damage Severity with Number of Event Types, *F*(3,99) = 3.24, *p* = .03, *η*^*2*^ = .09. This interaction replicates the results of Experiment 1, which were also consistent with our hypothesis that the additional requirement to track and integrate distinct types of evidence in a multifaceted problem would affect severity judgments, and confirmed our previous findings of attenuated severity judgments for multiple event types. This is illustrated by the shallower slopes of subjective severity ratings, as a function of differences in objective severity, for scenarios with two event types, *t*(33) = 2.18, *p* = .04. As in Experiment 1, planned contrasts showed that judgments differed significantly as a function of number of events for the relatively good scenarios, but not the relatively bad scenarios; *p* = .01, *p* = .04, *p* = .22, *p* = .80, respectively, for scenarios with 38%, 42%, 58%, and 63% of fruit damaged. At first glance, these four pairwise effects might suggest a trend in which the effect of Number of Event Types is strongest for the scenarios depicting problems with the lowest relative levels of severity. However, when we performed a paired samples t-test analogous to that in Experiment 1, the simple main effect of Number of Event Types did not differ significantly between the scenarios with the best crops (38% of fruit damaged) and those with the worst crops (63% of fruit damaged); *t*(33) = 1.68, *p* = .10. Instead, the consistent pattern across Experiments 1 and 2 is the shallower slope for scenarios with two event types. Results from the two experiments suggest that the effect of alternation can be driven by the need to integrate two event types over time. What, then, is the mechanism underlying the integration?

## Experiment 3

The goal of Experiment 3 was to examine a possible contributor to our previous findings where subjective severity judgments were attenuated for scenarios depicting two event types. One feature inherent to many multifaceted problems, including those examined here, is the random alternation between types of events signaling the problem. The rate of alternation in a sequence is known to affect attention to that sequence [9]. In addition, judgments can be biased by expectancies regarding the rate of alternation in a random sequence [10–12]. Expectancies regarding upcoming stimuli can in turn bias attention and influence subsequent judgments [13–17]. In light of these findings, we hypothesized that alternation between event types might bias judgments of problem severity. In order to explicitly test the effects of alternation, we held the Number of Event Types constant (always two) and manipulated the amount of alternation between types. In half of scenarios, event types were grouped into the first and last six years. In the other half of scenarios, event types alternated pseudo-randomly.

### Materials and methods

The methods of Experiment 3 were identical to those of Experiment 2 with the following exceptions.

#### Participants

Sixty-seven undergraduate university students participated in the experiment. Sixteen participants were excluded because their subjective ratings of damage severity demonstrated a failure to distinguish between objective differences. The remaining fifty-one participants (*N* = 36 females) had a mean age of 23.2 years (*SD* = 6.7). We used a larger sample than in Experiment 2 in order to obtain sufficient power to detect an interaction between our two independent variables at the *p* < .01. level.

#### Procedure

The procedure for Experiment 3 was identical to that of Experiment 2 with the following exceptions. There were no scenarios with only one event type. Instead, each scenario consisted of six years with insect damage and six years with mold damage. In place of manipulating the number of event types, we manipulated the ordering of event types. In the Ungrouped condition, the ordering of the two event types was pseudo-randomized in the same manner as in Experiments 1 and 2. In the Grouped condition, the scenario involved either six years with mold problems followed by six years with insect problems, or six years with insect problems followed by six years with mold problems. When matching scenarios across the Grouped and Ungrouped conditions in terms of the ordering of years with different severities of crop damage, the ordering was matched within each event type. For example, if the six years with insect problems followed the ordering: mild, mild, mild, moderate, severe in one scenario of a matched pair in the Grouped condition, then the years with insect problems would follow the same ordering in the other scenario of the pair, from the Ungrouped condition.

### Results and discussion

**Fig 4:**
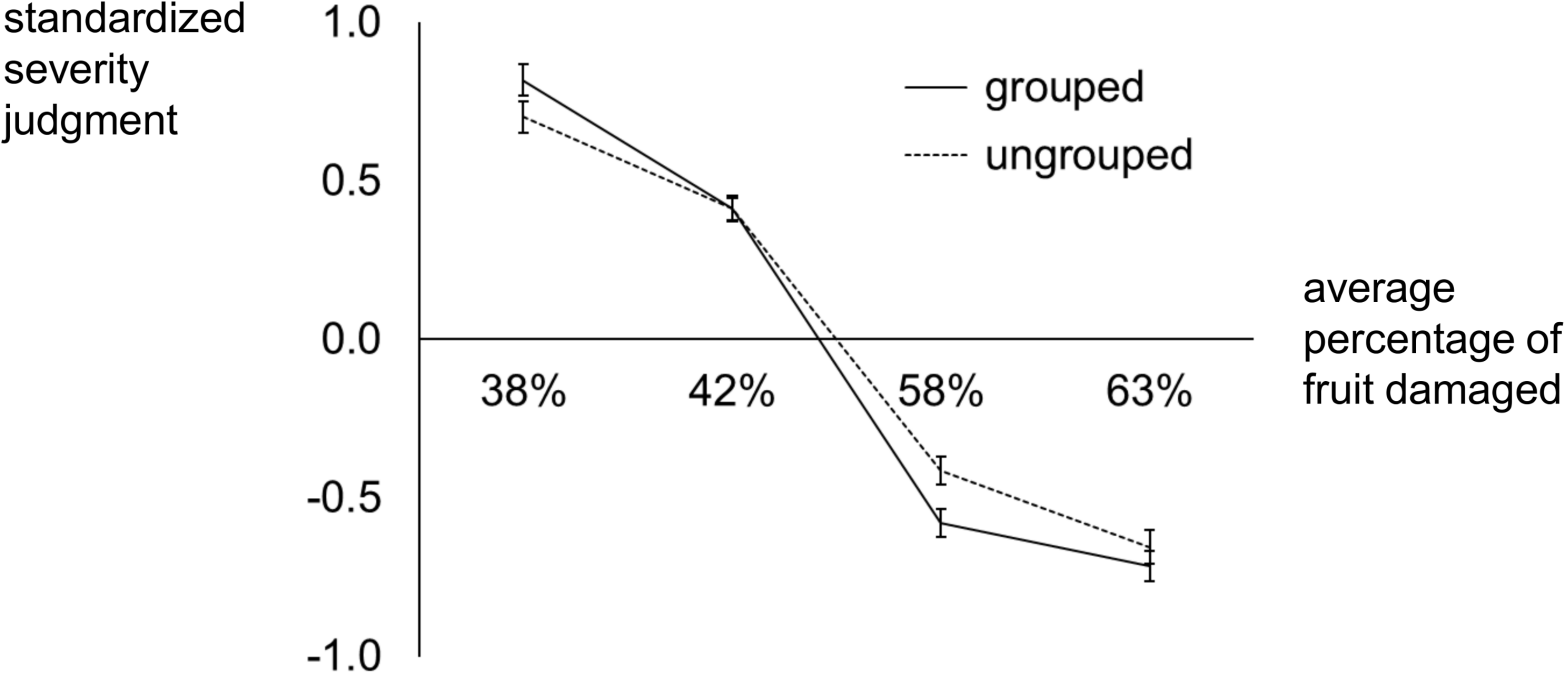
Experiment 3: Standardized subjective ratings of crop damage severity plotted as a function of objective crop damage severity (percentage of fruit damaged), plotted separately for scenarios involving pseudo-random alternation between the two event types and scenarios in which the two event types were organized into sequential groups of six (i.e. six years with insect damage followed by six years with mold damage, or vice versa).

The standardized subjective ratings of damage severity are plotted as a function of objective damage severity in Fig 4. We submitted these standardized ratings to a 2 × 2 repeated measures ANOVA with factors of Grouping (Grouped vs. Ungrouped) and Damage Severity. We found no significant main effect of Grouping, *F*(1,50) = 1.11, *p* = .30, *η*^*2*^ = .02. There was a main effect of Damage Severity, *F*(3,150) = 286.31, *p* < .001, *η*^*2*^ = .85, and an interaction of Damage Severity with Grouping, *F*(3,150) = 4.04, *p* = .012, *η*^*2*^ = .08. Subjective ratings of severity were attenuated (shallower slope) in the condition with alternating (ungrouped) event types, relative to the condition with grouped event types, *t*(50) = 2.26, *p* = .03. The shallower slope for alternating sequences in the current experiment is comparable to the shallower slope for scenarios depicting multiple event types in the previous two experiments. Planned contrasts of how judgments differed as a function of Grouping showed significance levels of *p* = .06, *p* = .98, *p* = .003, *p* = .34, respectively, for scenarios with 38%, 42%, 58%, and 63% of fruit damaged. This indicates that alternation between event types likely contributes to the attenuation of severity judgments for multifaceted problems observed in Experiments 1 and 2.

## General discussion

Here, we examined whether judgments of problem severity differed as a function of whether that problem was multifaceted. We used scenarios depicting damage to orchard crops by either two types of event (mold and insects) or one type. This allowed us to objectively quantify problem severity as the percentage of fruit damaged. The results of Experiments 1 and 2 showed that severity judgments were attenuated when problem severity was signaled by more than one type of evidence. Specifically, the slope of subjective severity ratings as a function of objective severity was shallower for scenarios with two event types than for those with only one event type. This was consistent with our hypothesis that the requirement to integrate across types of evidence would affect subjective severity judgments. In Experiment 3, we examined a possible contributor to the attenuation of severity judgments: the alternation between types of events signaling the problem. We found that the slope of subjective severity ratings plotted as a function of objective severity was shallower for scenarios involving frequent alternation between event types than for scenarios in which evidence was grouped into two successive longer streaks. In sum, these findings show that alternation between distinct types of evidence attenuates judgments of the severity of multifaceted problems.

The effect of attenuation is consistent with an interpretation whereby switching between categories increases cognitive demands. This raises interesting possibilities regarding the inherent cognitive load under the perception of alternating vs. repeating patterns [10–12]. One explanation is that it is more difficult to encode an alternating sequence than a repeating sequence in working memory [15]. A weaker memory representation of an alternating sequence might contribute to the attenuated judgments of severity. Another explanation is that a repeating sequence draws more attention than an alternating sequence, enhancing memory encoding of the regularities and consequently boosting the severity judgments of the repeating sequence [13]. The minimization of cognitive demands is a frequently invoked explanation of why individuals employ heuristics biasing attention to a subset of relevant evidence [18–21]. If evidence evaluation were more cognitively demanding for multifaceted problems, those demands could attenuate judgments of problem severity through differences in attention or working memory encoding.

Our findings also call for a revised interpretation of how evidence is processed in packed vs. unpacked form. In previous work [8], unpacking evidence into multiple *distinct events* while controlling for the number of evidence types *increases* how much that evidence affects judgments. Some of the earliest work on packing effects [19] becomes difficult to interpret as a result of the number of evidence types being confounded with the number of events considered. It is therefore important not only to control for evidence strength, but also to manipulate the types of unpacking (number of events vs. number of evidence types) independently. In our current studies, we demonstrate that unpacking evidence into multiple *distinct evidence types*, while controlling for the number of events, *decreases* how much that evidence affects judgments. Our findings are consistent with an interpretation whereby increasing the number of event types increases cognitive demands, weakening the representations of these events and thus perceived evidence strength.

One potential direction for future research relevant to the discussion of cognitive demands would involve studying framing effects in scenarios such as medical decision-making [22–24]. If positive vs. negative framing of a scenario (emphasizing gains vs. losses) were processed similarly to objectively good vs. bad scenarios, then we might expect alternation between event types to diminish the effects of both positive and negative framing, just as it diminishes the perceived severities of good and bad scenarios in the current study. A possible mechanism for such effects could involve increased cognitive load due to alternation between evidence types. The alternation may tax cognitive resources, in turn limiting the resources available for further processing. Future research could examine whether these findings interact with how the problem is framed [22–24]–whether we focus on the percentage of fruit damaged, as in the current study, or on the percentage of fruit saved. We might expect alternation between event types to diminish framing effects, which are reported to be stronger when conditions allow for substantive, effortful processing [23].

Dispositional optimism has been linked to biases in decision making [25]. Another potential direction for future research is to investigate whether under-estimation of event severity is associated with trait optimism [26]. Biologically grounded individual differences in behavioral activation and inhibition, which are indices of approach motivation and reward sensitivity [27], have also been indirectly linked to dispositional optimism [28]. A larger-sample study of individual differences could test the hypothesis that dispositional approach motivation predicts biases in severity judgments, particularly in more positive relative to more negative contexts. In sum, we find that judgments of severity are attenuated for multifaceted problems, relative to simpler problems. We also show that judgments of problem severity are attenuated if the distinct symptoms of the problem are ordered into sequential groups (long streaks) rather than alternating more frequently. This suggests that alternation between evidence types may contribute to the attenuation of severity judgments for multifaceted problems. These findings have broad relevance for recognizing changes in ‘real-world’ types of problems, including illnesses with a variety of symptoms, economic trends with broad effects, and environmental problems such as climate change leading to a range of severe events (e.g. floods, droughts, storms and fires).
